# Clinical and survival outcomes of colectomy for transverse colon cancer in elderly patients

**DOI:** 10.1097/MD.0000000000033046

**Published:** 2023-03-03

**Authors:** Xiang-Jun Liu, Zhi-Quan Lang, Wei Zhang, Xiao-Qing Zhang, Ping-Fan Lu, Feng Xie, Bo Liang, Zhi-Ping Huang, Zhen-Hong Zou

**Affiliations:** a Department of General Surgery, The Second Affiliated Hospital of Nanchang University, Jiangxi, People’s Republic of China; b Zhongnan Hospital of Wuhan University, Institute of Hepatobiliary Diseases of Wuhan University, Transplant Center of Wuhan University, Hubei Key Laboratory of Medical Technology on Transplantation, Wuhan, Hubei, People’s Republic of China; c Departments of Hepatobiliary Surgery, General Hospital of Southern Theatre Command, Guangzhou, People’s Republic of China.

**Keywords:** elderly patients, prognosis, transverse colon cancer

## Abstract

It remains controversial whether elderly patients with transverse colon cancer present worse prognoses. Our study utilized evidence from multi-center databases to evaluate the perioperative and oncology outcomes of radical resection of colon cancer in elderly and nonelderly patients. In this study, we analyzed 416 patients with transverse colon cancer who underwent radical surgery from January 2004 to May 2017, including 151 elderly (aged ≥ 65 years) and 265 nonelderly (aged < 65 years) patients. We retrospectively compared the perioperative and oncological outcomes between these 2 groups. The median follow-up in the elderly and nonelderly groups was 52 and 64 months, respectively. There were no significant differences in the overall survival (OS) (*P* = .300) and disease-free survival (DFS) (*P* = .380) between the elderly and nonelderly groups. However, the elderly group had longer hospital stays (*P* < .001), a higher complication rate (*P* = .027), and fewer lymph nodes harvested (*P* = .002). The N classification and differentiation were significantly associated with OS based on univariate analysis, and the N classification was an independent prognostic factor for OS based on multivariate analysis (*P* < .05). Similarly, the N classification and differentiation were significantly correlated with the DFS based on univariate analysis. However, multivariate analysis indicated that the N classification was an independent prognostic factor for DFS (*P* < .05). In conclusion, the survival and surgical outcomes in elderly patients were similar to nonelderly patients. The N classification was an independent factor for OS and DFS. Even though elderly patients with transverse colon cancer present a higher surgical risk than nonelderly patients, performing radical resection in elderly patients can be an appropriate choice for treatment.

## 1. Introduction

Colorectal cancer is the 4th most common cancer globally, increasing the financial burden on cancer patients and society. Colorectal cancer is the second leading cause of cancer-related deaths in the West, severely threatening people’s lives and health.^[1]^ Transverse colon cancer occurs between the liver and spleen in the curved part of the colon. Only 10% of colorectal cancer cases occur in the transverse colon, which is a relatively low cancer incidence.^[2,3]^

In recent years, an increasing number of retrospective studies have shown that laparoscopic resection is a safe and effective treatment for transverse colon cancer.^[4–16]^ Previous studies have demonstrated that laparoscopic colectomy can achieve similar results in elderly and nonelderly patients with colon cancer.^[17–22]^ Since average life expectancy in China has increased gradually, the incidence of colon cancer in the elderly has increased correspondingly. However, the relationship between aging and the clinical outcome of transverse colon cancer remains unclear. Most patients diagnosed with colon cancer are between 50 and 70 years, and recently, the incidence of colorectal cancer among young people has been increasing, but the survival rate remains low.^[23,24]^ In addition, few studies have focused on investigating transverse colon cancer due to its low incidence compared to other forms of colon cancer. In addition, few published studies have reported long-term results on tumors in patients, especially for transverse colon cancer. Even fewer research reports have explicitly focused on transverse colon cancer. Therefore, it is necessary to better understand the results of long-term oncology treatment of transverse colon cancer.

We used a multi-center approach comparing perioperative and oncological prognoses to evaluate the hypothesis that the survival rates and surgical outcomes of elderly patients were similar to nonelderly patients with transverse colectomy, even though elderly patients presented a higher surgical risk. Therefore, this study compared the colectomy survival rates and surgical outcomes of elderly and nonelderly patients undergoing transverse colectomy.

## 2. Materials and Methods

Data were collected on patients who underwent transverse colon cancer resection in the 3 affiliated hospitals of Nanchang University from January 2004 to May 2017. We retrospectively analyzed patients with a pathological diagnosis of transverse colon cancer based on the 7th edition of the American joint committee on cancer staging system and using the existing databases. We assessed the patient’s case information, postoperative complications, and overall survival and disease-free survival rates. This study adhered to the principles contained in the Declaration of Helsinki. In addition, the ethics committee of the Second Affiliated Hospital of Nanchang University approved the study.

### 2.1. Surgical indications and procedures

Before treatment, the combination of endoscopic biopsy and abdominal computed tomography was typically used for preoperative diagnosis and establishment of the clinical stage of the disease. For tumors located in the curvature of the liver, right hemicolectomy was performed, for tumors located in the curvature of the spleen, and left hemicolectomy was performed. When carrying out a right hemicolectomy, the origins of the right colic, ileocolic, the right branch of the middle colic vessels were ligated, and the associated lymph nodes were dissected. The origin of the midgut colic blood vessel was ligated when an enlarged right hemicolectomy was performed. The protocol associated with a left colectomy was to ligate the origin of the left branches of the left and middle colic blood vessels as well as dissection of the associated lymph nodes. For an enlarged left colectomy, the origins of the colic vessels in the midgut were ligated. Transverse colectomy was accomplished by ligating the blood vessels in the middle of the colon and dissection of associated lymph nodes. The surgeon decided which surgical method was used. The surgeries were performed based on the principle of complete mesosphere resection.^[25]^ The transition from laparoscopic surgery to open surgery was defined as an incision larger than needed for specimen retrieval or the need to make an unplanned abdominal incision.

### 2.2. Follow-up

Patients were followed after surgery every 3 months for the first 2 years. Then follow-up occurred every 6 months until the fifth year after surgery. After 5 years, a follow-up visit was scheduled once a year. Follow-up information was collected using telephone interviews or door-to-door visits, as well as examining medical records.

### 2.3. Statistical analysis

The data were presented as means ± standard deviation to represent continuous variables that conformed to a normal distribution or as a median (range) and a value (%) that represented a categorical variable. The Kaplan–Meier method was used to estimate overall survival (OS) and disease-free survival (DFS). Univariate and multivariate analyses were conducted using the Cox model. The results were reported as risk hazard ratio (HR) with a 95% confidence interval (95% CI). A 2-sided *P* value < .05 was considered statistically significant. The statistical analyses were performed using SPSS (version 21.0; SPSS Inc., Chicago, Illinois).

## 3. Results

**Figure 1.**
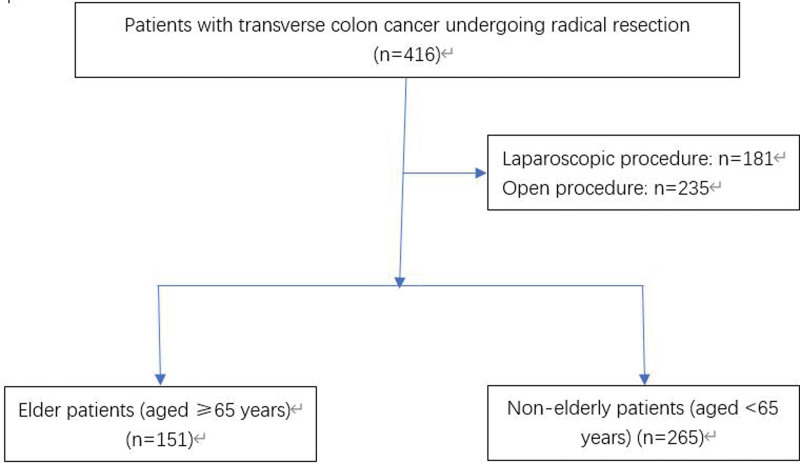
A flow chart showing the study protocol.

The current study included a total of 416 cases that received radical resection of the transverse colon; 151 cases (36.3%) were elderly patients, 265 cases (63.7%) were nonelderly patients, as shown in Figure 1. After comparing the preoperative data between the 2 groups, the scores of the elderly group and the American College of Anesthesiologists were higher than the nonelderly group. Except for the ASA score, the age, gender, clinical stage (7th American joint committee on cancer-UICC), and previous abdominal surgery history were not significantly different, as shown in Table 1.

**Table 1:** Baseline characteristics of the 2 groups.

Characteristics	Elderly group (n = 151)	Nonelderly group (n = 265)	*P* value
Age, yr, median (range)	71 (65–86)	52 (15–64)	.000
Gender			.523
Male	80	141	
Female	71	124	
ASA score, patients (n)			.001
1	15	37	
2	108	212	
3	27	15	
4	1	1	
Clinical stage (7th AJCC-UICC)			.936
I	6	12	
II	100	170	
III	45	83	
Abdominal surgery history			.055
Present	25	28	
Absent	126	237	

AJCC = American joint committee on cancer.

The operative and postoperative outcomes of the 2 groups are presented in Table 2. Elderly patients with transverse colon cancer exhibited longer hospital stays (13 vs 12 days, *P* = .027) and a higher postoperative complication rate (37.7% vs 24.9%, *P* < .001). However, the postoperative mortality was comparable between the 2 groups. There were no significant differences in the surgical procedures used (laparoscopic vs open), surgery time, blood loss, time to first flatulence, and time to return to a liquid diet (Table 2).

**Table 2:** Operative and postoperative outcomes of the 2 groups.

Outcomes	Elderly group (n = 151)	Nonelderly group (n = 265)	*P* value
Type of resection			.102
Right hemicolectomy	101	167	
Left hemicolectomy	21	58	
Transverse colectomy	29	40	
Procedures (laproscopic vs open)	67:84	114:151	.434
Operative time, min, median (range)	180 (85–370)	180 (83–510)	.644
Blood loss (mL), median (range)	150 (10–3500)	150 (20–1000)	.818
Time to pass first flatus (d)	4 (1–10)	4 (1–11)	.910
Time to resume liquid diet (d), median (range)	5 (2–13)	5 (1–46)	.586
Hospitalization (d), median (range)	13 (6–42)	12 (1–50)	.027
Patients with postoperative complications (n)	57	66	<.001
Postoperative mortality (n)	1	2	.701

**Figure 2.**
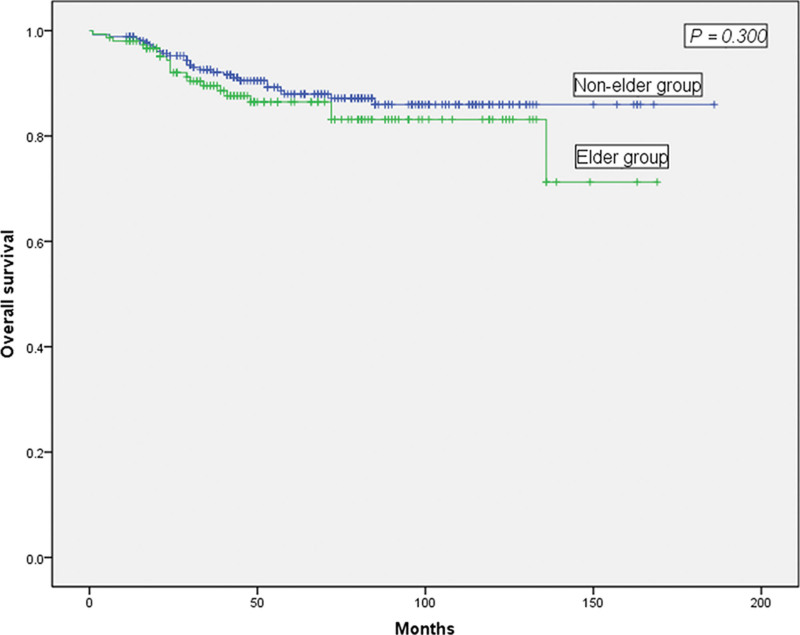
Comparison of the overall survival rate between elderly (aged ≥ 65 years) and nonelderly (aged < 65 years) patients. The 2 groups were not significantly different (*P* = .300).

**Figure 3.**
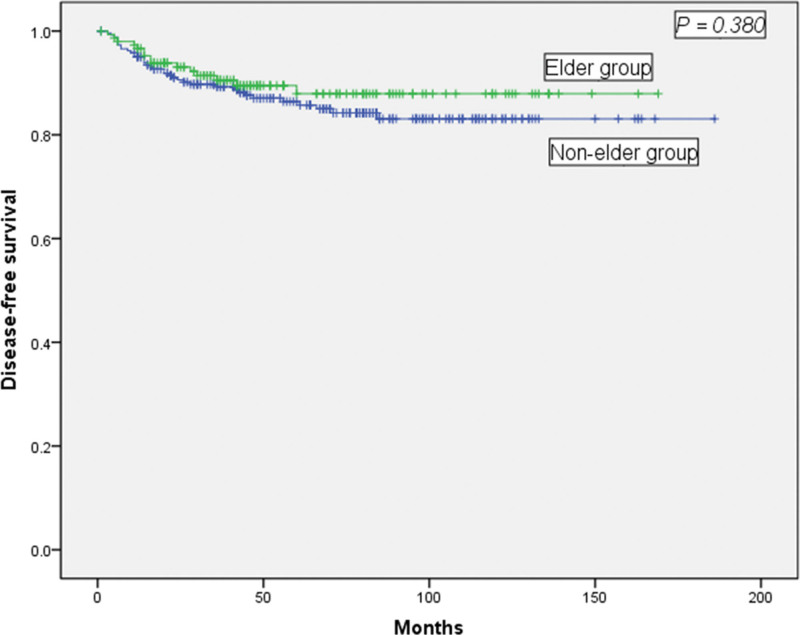
Comparison of the disease-free survival rate between elderly (aged ≥ 65 years) and nonelderly (aged < 65 years) patients. No significant difference was observed (*P* = .380).

The 2 groups exhibited similar tumor histological differentiation and tumor sizes based on the pathological examinations. However, a statistically smaller number of lymph nodes were harvested in the elderly group (11 vs 13, *P* = .002) (Table 3). The average follow-up times for the 2 groups were 52 months and 64 months, respectively (*P* = .191), and there was no significant difference in the OS or DFS between the 2 groups. As shown in Figure 2, the 5-year OS rates for the elderly and nonelderly groups were 88.6% and 88.0% (Fig. 2, *P* = .300), respectively. The 5-year DFS rates for the elderly and nonelderly groups were 90.5% and 89.7%, respectively, which were not significantly different (Fig. 3, *P* = .380).

**Table 3:** Pathological outcomes of the 2 groups.

Outcomes	Elderly group (n = 151)	Nonelderly group (n = 265)	*P* value
Tumor differentiation			.249
Well	5	5	
Moderate	123	210	
Poor	23	50	
Tumor size (cm), median (range)	5 (1–18)	5 (2–15)	.387
Harvested lymph nodes, median (range)	11 (0–40)	13 (0–74)	.002

As shown in Table 4, univariate analysis revealed that the N classification (HR = 5.814, 95% CI: 2.004–16.949) and differentiation (HR = 2.498, 95% CI: 1.409–4.429) presented significant associations with OS. Multivariate analysis indicated that the N classification (HR = 8.403, 95% CI: 1.709–41.667) was an independent prognostic factor for OS. Similarly, the N classification (HR = 7.936, 95% CI: 3.030–20.833) and differentiation (HR = 2.385, 95% CI: 1.355–4.200) were significantly correlated with DFS based on univariate analysis. Furthermore, multivariate analysis indicated that the N classification (HR = 19.608, 95% CI: 4.504–83.333) was an independent prognostic factor for DFS.

**Table 4:** Prognostic factors for overall and disease-free survival (n = 416).

	OS					DFS			
	Univariate analysis	*P*	Multivariate analysis	*P*	Univariate analysis		*P*	Multivariate analysis	*P*
	HR (95% CI)		HR (95% CI)		HR (95% CI)			HR (95% CI)	
Age (yr), ≥60 <60	1.153 (0.762–2.404)	.302	1.677 (0.743-3.787)	.213	0.764 (0.418–1.397)		.382	0.855 (0.372–1.967)	.713
Gender, male: female	1.044 (0.592–1.843)	.882	1.648 (0.723-3.757)	.235	0.998 (0.576–1.729)		.994	1.286 (0.591–2.795)	.526
T stage, T1 & T2: T3 & T4	0.673 (0.084–5.388)	.320	0.394 (0.099-1.572)	.879	0.734 (0.090–5.972)		.575	0.240 (0.051–1.124)	.611
N stage, N0: N1 & N2	5.814 (2.004–16.949)	˂.001	8.403 (1.709-41.667)	.005	7.936 (3.030–20.833)		˂.001	19.608 (4.504–83.333)	.002
Tumor size (cm), ≥6: <6	1.055 (0.591–1.882)	.865	1.231 (0.489-3.097)	.659	0.981 (0.562–1.713)		.946	1.105 (0.488–2.501)	.810
Cancer nodules, positive: negative	1.227 (0.967–1.557)	.092	1.113 (0.796-1.557)	.531	1.166 (0.927–1.466)		.189	0.979 (0.676–1.427)	.910
Differentiation, well: moderate: poor	2.498 (1.409–4.429)	.002	2.112 (0.794-5.619)	.134	2.385 (1.355–4.200)		.003	1.899 (0.769–4.694)	.165
ASA, 1:2:3	1.386 (0.784–2.452)	.261	1.134 (0.512-2.511)	.756	1.081 (0.615–1.897)		.787	0.953 (0.437–2.079)	.904
Procedures, open: laparoscopic	1.344 (0.716–2.523)	.358	1.616 (0.644-4.054)	.307	1.048 (0.590–1.859)		.873	1.217 (0.543–2.727)	.634

95% CI = 95% confidence interval, DFS = disease-free survival, HR = hazard ratio, OS = overall survival.

## 4. Discussion

Transverse colon cancer occurs between the liver and the splenic flexure of the colon, and it occurs in only 10% of colorectal cancer patients.^[2,3]^ Several retrospective reports have shown that compared to open colectomy, elderly patients with colon cancer undergoing laparoscopic colectomy could achieve improved surgical outcomes and similar survival results.^[17–22,26]^ However, these previous trials did not include malignant tumors of the transverse colon. There were several possible reasons for this omission. First, the anatomical structures adjacent to the transverse colon are complicated, making the surgical anatomy challenging. Second, removing malignant tumors associated with the transverse colon necessitates excellent surgical skills. The anatomical changes in the blood vessels at the mid-colon require exceptional technical skills for lymph node dissection. Finally, because only 10% of colon malignancies are found in the transverse colon, surgeons have limited experience carrying out surgeries in this area.

This study retrospectively analyzed 416 cases of transverse colon cancer that included elderly and nonelderly patients and explored the prognosis and clinical results of patients who underwent radical surgery. The median follow-up times were 52 and 64 months, the 5-year OS rates were 88.6% and 88.0%, and the 5-year DFS rates were 90.5% and 89.7%, and respectively, for the elderly and nonelderly groups. The N classification was an independent factor for OS and DFS. However, no significant difference was observed for OS and DFS between the elderly and nonelderly groups. These observations were similar to previous studies.^[27]^ The data reveal that elderly and nonelderly patients with transverse colon cancer exhibited similar overall survival and disease-free survival rates. Possible reasons for these outcomes include vague symptoms, failure to seek medical attention, and misdiagnosing benign versus malignant tumors in nonelderly patients. In addition, young patients are often diagnosed as having undifferentiated or poorly differentiated colorectal cancer at a relatively late stage based on Duke staging system for colorectal cancer, reducing the possibility of successful surgical treatment.^[28,29]^ These factors could lead to survival rates that are similar to those observed for elderly patients. Thus, clinicians should pay more attention to young patients and change their traditional diagnostic methods to increase the possibility of early diagnosis and treatment, which could improve the prognosis of young colorectal cancer patients. On the other hand, advanced age, lower tolerance to surgery and chemotherapy, a higher incidence of complications, and postoperative complications serve to decrease the prognoses of elderly colorectal cancer patients. Therefore, these reasons might explain the lack of any significant differences in the OS and DFS between elderly and nonelderly patients.

Notably, the previous studies are controversial with respect to whether age affects the clinical outcomes and parameters of patients with transverse colon cancer.^[19,28]^ These conclusions might be due to an unclear status of transverse colon cancer in some patients or the relatively small sample size. Therefore, a larger sample size and higher quality prospective studies are needed to confirm this finding.

Our study revealed that when comparing the OS and DFS between the elderly and nonelderly groups, the elderly group exhibited longer hospital stays (*P* < .000), a higher complication rate (*P* = .027), and fewer lymph nodes that were sampled (*P* = .002). Therefore, this study demonstrated that for transverse colon resection, older patients could experience similar long-term outcomes as nonelderly patients. Furthermore, greater attention should be focused on increasing postoperative treatment and care of elderly patients.

Several limiting factors were associated with this study. First, although the sample size of the surgical resections of transverse colon malignancies in this study was larger than in previous surveys, the incidence of transverse colon malignancies only accounted for 10% of colorectal cancers, limiting the opportunity for additional randomized controlled trials that would allow more conclusive results. Second, although this was a multi-center retrospective study, sample selection bias still could have occurred. Despite these limitations, these results supported radical surgery for transverse colon cancer in elderly and nonelderly patients. It is necessary to conduct higher quality prospective studies with larger sample sizes to more accurately determine the impact of age on the effectiveness and safety of treatment for patients with transverse colon cancer.

## 5. Conclusion

This investigation demonstrated that the survival and surgical outcomes in elderly patients were similar to nonelderly patients. Although patients with transverse colon cancers have higher surgical risk than nonelderly patients, radical resection in elderly patients could be a viable choice to treat transverse colon cancer.

## Acknowledgments

The authors would like to express their gratitude to EditSprings (https://www.editsprings.cn) for the expert linguistic services provided.

## Author contributions

**Conceptualization:** Ping-Fan Lu, Zhiping Huang, Zhenhong Zou.

**Data curation:** Xiang-Jun Liu, Ping-Fan Lu.

**Formal analysis:** Ping-Fan Lu.

**Funding acquisition:** Zhi-Quan Lang, Feng Xie.

**Investigation:** Zhi-Quan Lang.

**Methodology:** Xiang-Jun Liu, Zhi-Quan Lang, Feng Xie, Zhenhong Zou.

**Project administration:** Zhi-Quan Lang.

**Resources:** Zhi-Quan Lang.

**Software:** Zhi-Quan Lang.

**Supervision:** Ping-Fan Lu, Zhenhong Zou.

**Validation:** Zhiping Huang, Zhenhong Zou.

**Writing – original draft:** Xiang-Jun Liu.

**Writing – review & editing:** Xiang-Jun Liu, Wei Zhang, Xiao-Qing Zhang, Feng Xie, BO Liang.

## Correction

When originally published, this article listed an incorrect author as the corresonding author. Zhi-Ping Huang has been replaced with Zhen-Hong Zou as the corresponding author.
